# Eco Friendly Synthesis of Carbon Dot by Hydrothermal Method for Metal Ions Salt Identification

**DOI:** 10.3390/ma14247604

**Published:** 2021-12-10

**Authors:** Hasan Shabbir, Tomasz Tokarski, Ditta Ungor, Marek Wojnicki

**Affiliations:** 1 Faculty of Non-Ferrous Metals, AGH University of Science and Technology, Mickiewicza Ave. 30, 30-059 Krakow, Poland; marekw@agh.edu.pl; 2 Academic Centre for Materials and Nanotechnology, AGH University of Science and Technology, Al. A. Mickiewicza 30, 30-059 Krakow, Poland; tokarski@agh.edu.pl; 3 MTA-SZTE “Lendület” Noble Metal Nanostructures Research Group, University of Szeged, Rerrich B. Square. 1, H-6720 Szeged, Hungary; ungord@chem.u-szeged.hu

**Keywords:** carbon quantum dots, low-cost and one-step synthesis, hydrothermal reaction, temperature and time effect, photoluminescence, metal ions sensing, limit of detection and quantification

## Abstract

In this work, we report the synthesis method of carbon quantum dots (CDs) using the one-step method for fast and effective metal ion determination. Ascorbic acid was used as an inexpensive and environmentally friendly precursor. High-pressure and high-temperature reactors were used for this purpose. Microscopic characterization revealed the size of CDs was in the range of 2–6 nm and they had an ordered structure. The photoluminescence properties of the CDs depend on the process temperature, and we obtained the highest PL spectra for 6 h of hydrothermal reaction. The maximum emission spectra depend poorly on synthesis time. Further characterization shows that CDs are a good contender for sensing Fe^3+^ in aqueous systems and can detect concentrations up to 0.49 ppm. The emission spectra efficiency was enhanced by up to 200% with synthesis time.

## 1. Introduction

Carbon quantum dots (CDs) are a new class of nanomaterial discovered in 2006, with a size below 10 nm and a wide range of fascinating properties [1] CDs consist of carbon, oxygen, hydrogen, and nitrogen, and sp^2^-hybridized conjugating bonding are dominant while sp^3^-hybridized bonding is also present [2]. Because of their unique photoluminescence (PL) properties, CDs have sparked a lot of interest in the research community due to intense and tunable fluorescence around the visible range, which also contradicts the traditional view of carbon as a black material unable to emit light, and regarding its surface activity, and small size. They are also interesting materials in terms of biocompatibility, low toxicity, high water solubility, environment-sensitive characteristics, and electrochemical luminescence. These properties are the reason for the potential application of CDs in different fields including biosensing, drug-delivery, bioimaging, photocatalysis, light-emitting devices [3,4], and so on. They have comparable fluorescent properties with semiconductor quantum dots but have some advantages over them, such as biocompatibility, easy synthesis procedure, and high resistance to photobleaching [5]. As a result, there has been a surge in research into this kind of nanomaterial production. Eco-friendly, often known as green synthesis, is gaining attention. The eco-friendly synthesis method produces minimal pollutants, uses nontoxic solvents, and produces no side waste with the final product, resulting in a finished product that is environmentally friendly. An eco-friendly synthesis method is necessary to avoid the generation of undesirable or dangerous by-products by developing dependable, long-term, and environmentally friendly synthesis techniques [6]. The use of nontoxic solvents and precursors, which are largely organic chemicals, is critical for achieving eco-friendly synthesis, which we will investigate in this study [7].

Most of the CDs are synthesized via two different approaches: top-down and bottom-up methods [8]. In the top-down method, larger graphite materials are sliced into small carbonaceous nanomaterials including CDs by physical and chemical reactions. The top-down methods include chemical oxidation, electrochemical oxidation, laser ablation, etc. [9,10,11]. In the bottom-up method, the approach is vice versa and includes thermal decomposition, hydrothermal methods, and microwave synthesis [11,12,13,14,15]. Hydrothermal synthesis is considered more fascinating and practical due to its low cost, nontoxicity, environmentally friendly nature, and high quantum yield. There are a lot of different natural precursors reported for CDs synthesis, including banana juice [16] and pulp [17], orange juice [18], sago waste [2], chia seeds [19], mango peel [20], potato starch [21], and waste paper [22].

Because CDs are primarily composed of carbon, they can be used in applications where toxic heavy metals such as Hg, Pb, and Cd are not appropriate [23]. To make them more viable for use in real-world applications, the quantum yield and product yield should be improved. Different synthetic approaches report a quantum yield of less than 10% and very small amounts of the final product, generally less than 10 mg^3^.

Here, in this research, we reported the synthesis of CDs from ascorbic acid using a high-pressure, high-temperature microwave reactor. These CDs can be used to detect different metal ions with single-step fluorescence spectroscopy. The product yield is about 3 g of CDs for 20 mL of the initial solution.

Some heavy metals, such as iron and zinc, are required for human metabolism and are rarely hazardous to human health when present in adequate amounts. Other metals, such as Pb^2+^, and Cd^2+^, on the other hand, are toxic to humans even in small concentrations [24]. Iron (Fe^3+^) is the most significant transition metal in organisms and biological systems, with important functions in oxygen transport and exchange, as well as enzymatic reactions at the cellular level. Additionally, iron in water promotes the growth of iron bacteria, which produces slime that can stain and block the plumbing of arteries [25,26]. As a result, due to growing worries about industrial pollution, the detection of ions of this transition metal is highly wanted. Current methods for determining metal ions in water need time, skill, and controlled laboratory settings, and techniques such as inductively coupled plasma mass spectrometry, atomic fluorescence spectroscopy, or atomic absorption spectroscopy are commonly used for detection [27]. Iron is also produced during hydrometallurgy of zinc, and more than 90% of zinc produced by hydrometallurgy. The zinc sulphide concentrate is roasted, and the resulting zinc calcine is leached into two phases, resulting in iron impurities in the leach liquid that cannot be avoided. To reduce the amount of iron it is necessary to detect its amount [28]. Due to these constraints, affordable fluorescence probing is becoming more popular as an analytical technique for detecting iron due to its sensitivity, selectivity, and speed. So, in the current work, our focus is to detect Fe^3+^ by eco-friendly CDs that do not require high proficiency of expertise to work with [29]. Herein we reported the limit of detection and quantification of Fe^3+^ with CDs.

CDs are a suitable candidate component for iron sensors in this regard because they are biocompatible. Furthermore, the fluorescence quantum yield of CDs can be improved by altering the surface groups and intrinsic components on the CDs surface [30]. Understanding the structure of CDs is crucial to comprehending their key characteristics, such as fluorescence. The graphitic interlayer gaps of 0.32 nm and graphitic in-plane lattice spacing on CDs is typically 0.18–0.24 nm [24,31]. We compare the detection of several types of salts, both toxic and nontoxic, with Fe^3+^ in this work. 

## 2. Experimental and Materials

A hydrothermal treatment method was used to obtain CDs. In the typical experiment, 2.0 g of (purity in the range of 99–100%) ascorbic acid (0.55 molar solution) (Avantor Performance Materials, Gliwice, Poland) was dissolved in 20 mL of deionized water (Polwater, conductivity < 0.05 µS), and we carried out the reaction in the temperature range 180–190 °C for different durations: 1, 2, 4, 6 and 8 h. A specialized reactor Ertec Magnum II was used (Ertec Poland, Wroclaw, Poland). Magnum II has a maximum filling capacity of 108 cm^3^. Maximum microwave output power is equal to 600 W. The maximum attainable temperature is 300 °C and the maximum pressure is 50 bars [32]. In this apparatus, it is also possible to monitor the temperature and pressure during the reaction. The experiment was repeated twice for 6 h, with temperature variation and for 160 °C and 240 °C. A light-yellow coloured suspension was obtained. The dialysis method was applied to remove the particles of larger size and to obtain the colloidal solution. The dialysis lasted for one hour, and the dialysis tube had the following specifications: wall thickness 0.03 mm, diameter 28.6 mm, and length 30 m.

The PL spectra of the CDs were registered on an Applied photophysics spectrofluorometer (Applied Photophysics Ltd, Leatherhead, United Kingdom) in a standard quartz cuvette with 1 cm optical length, applying a 1000-fold dilution in ultrapure water. The instrument was equipped with a WI calibrated light source and the quantum yield (QY%) was measured in an integrated sphere with 100 mm diameter. For the calculation, 0.5 mg/mL Rhodamine B in ethylene glycol solution was used as a reference. The spectra were analysed with the JASCO Spectra Manager 2.0 program. L-Ascorbic acid used as fine powder in this experiment was obtained from Avantor Performance Materials Poland. After synthesis, the solution was filtered using a 90 mm, quantitative hard, ash-free, reinforced paper filter.

UV–Vis spectra were measured using the Shimadzu model U–2501 PC spectrophotometer (Shimadzu corporation, Kyoto, Japan), working in the wavelength range of 190–900 nm. The baseline was corrected against the reference water-filled quartz cuvette. Spectra were recorded using a slight 1 nm and standard 1 cm quartz cuvette. HR-TEM analysis was performed using the instrument (HR–TEM)–FEI TECNAI TF 20 X-TWIN (Thermo Fisher Scientific, Bremen, Germany). For this purpose, one drop of freshly prepared colloidal suspension was placed on a copper grid covered by a 20–30 nm amorphous carbon film. Then, the samples were left to dry at room temperature. We used a method described in the literature to reduce the amount of unwanted organic ligands produced during CDs synthesis, thereby improving TEM scanning image quality [33]. In practice, the optimized method based on four steps using ethanol and activated carbon is very simple to implement. Activated carbon is placed in a container and then ethanol is poured into the container to completely cover the activated carbon. Many bubbles rise from the activated carbon. A graphitic grid of TEM is inserted into the container to let the bubbles pass through the grid, and it is held for 1 min with tweezers. Then the grid is left to dry for 4 h and used for microscopy.

The zeta potential of the obtained CDs, as well as the size and size distribution, were determined using the Zetasizer Nano ZS (Malvern Instrument, Malvern, United Kingdom). For this purpose, a standard clear polycarbonate cell with gold electrodes was used. Fluorescence spectra were measured using a Photophysics SX20—Spectrometer (Applied Photophysics Ltd, Leatherhead, United Kingdom) using a 1 cm, 4-wall transparent quartz cuvette.

The FT-IR spectra were registered using Nicolet 380 spectrometer (Thermo Fisher Scientific, Bremen, Germany). CDs suspensions were mixed with potassium bromide powder of spectroscopic grade and dried for 24 h at 353 K. The base pellets, as well as samples, were formed using a hydraulic press. For this purpose, a 13 mm in diameter mould was used. For the samples to be identical, 0.2 g KBr ± 0.0002 g was used in each case.

XRD analysis was performed using Rigaku miniflex diffractometer (Rigaku Corporation, Tokyo, Japan). Two analyses were performed. The first analysis was carried out for the sample holder (made of glass) used for analyses. The second analysis was performed on a sample where the QDs were spotted on the sample holder, then evaporated at 60 °C.

## 3. Results

### 3.1. Optical Properties of CDs

**Figure 1:**
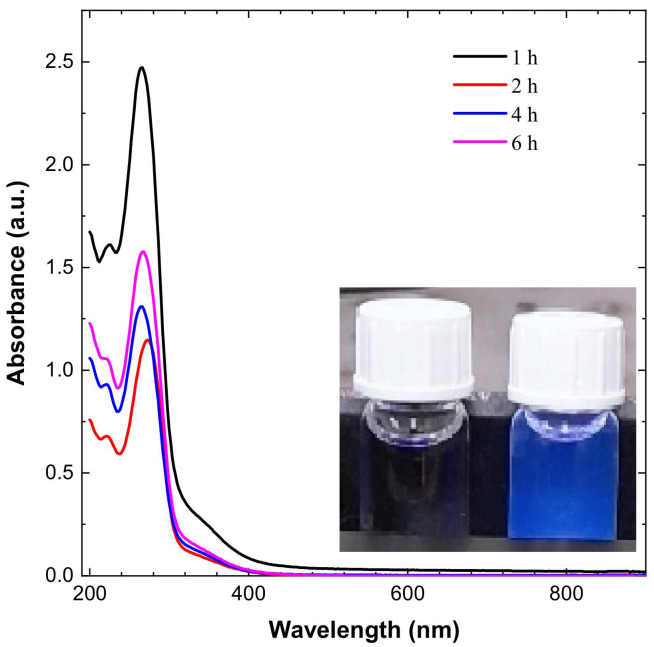
UV spectroscopy results for CDs and emission of blue light from CDs on exposure to UV irradiation.

The CDs’ dispersion appeared pale yellow in sunlight and blue when exposed to UV irradiation, as shown in Figure 1. The CDs have an absorbance peak in the range of 267 to 280 nm, which is expanding into visible range, which is attributed to π–π * transition of conjugated C=C and noncovalent weak n–π * transition of C=O bonds, respectively, as shown in Figure 1. 

The peak shifted from 267 to 280 nm for different durations of time, and has the value of 280 nm for a 2 h reaction. The UV–Vis graph shows that maximum absorbance is variable with time and varies from 267 nm to 280 nm [34,35].

**Figure 2:**
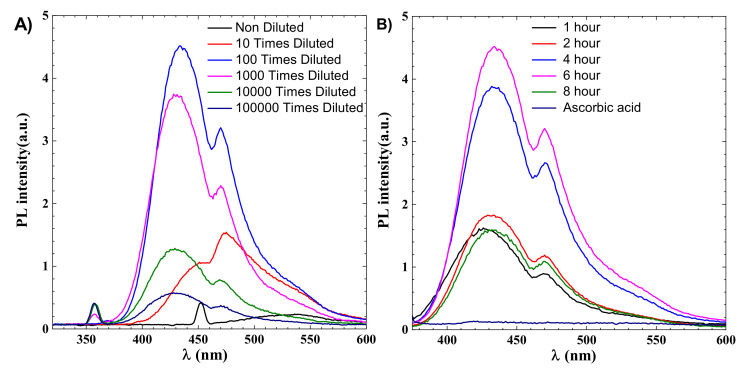
(**A**) Emission spectroscopy of CDs for 6 h, the effect of dilution on intensity (**B**) effect of synthesis time on intensity.

CDs are identified by their emission wavelength and size-dependent photoluminescent (PL) behavior. PL is one of the most fascinating CDs behaviours, both fundamentally and practically. The intensity of the PL peak increases with a decrease in the concentration of CDs, as shown in Figure 2. This can be explained due to decreased interactions between the various polar functional groups at low concentrations [36]. At high concentrations, the presence of polar functionality aids in the formation of agglomeration. After dilution up to 10,000 times, CDs’ effect becomes less dominant, as shown in Figure 2A. PL phenomena in CDs have not been fully explainable until now due to their complex behaviour, but the distribution of the different surface energy traps and variation in particle size of CDs is considered to be the main reason behind the PL behaviour of CDs. There is also a quantum confinement effect in CDs due to lower size, which also contributed to their features, and with an decrease in the size of CDs the bandgap increases. The surface functional groups can also act as an emission trap for π–π * transition of conjugated C=C, so surface defects and CDs size are considered the two main reasons for the PL mechanism. The optimized fluorescence intensity is achieved for a 6 h reaction, as shown in Figure 2B.

As shown in Figure 2B, the maximum fluorescence intensity for 6 h is 4.4, which is more than twice the 8 h reaction intensity which is around 1.5, indicating that synthesis time is an important parameter for industrial applications that require photoluminescence. From Figure 2B it can be noted that PL intensity increases with time and decreases sharply after 6 h, and the emission spectra of 1 and 8 h are almost the same at nearly 1.5, indicating 6 h as the optimized time for hydrothermal reaction.

**Figure 3:**
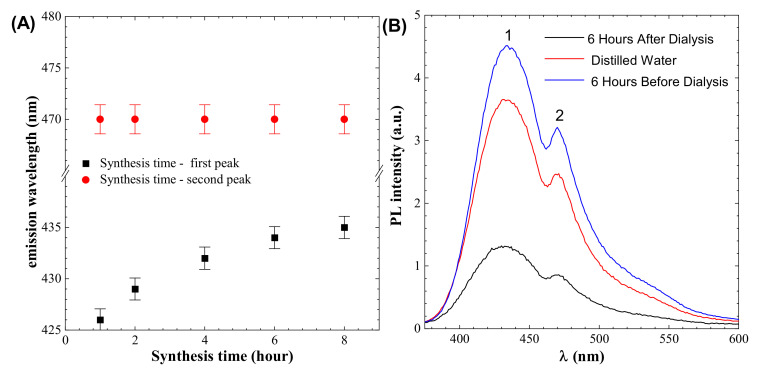
(**A**) Effect of synthesis time on peaks (**B**) dialysis effect on the colloidal solution (Peak 1 indicates emission peaks that are depending on synthesis time, whereas peak 2 is independent by synthesis time.).

PL spectroscopy shows two peaks. One is near 433 nm, which represents the emission of light, and this peak position changes with synthesis time, as shown in Figure 3A. The second peak is at 470 nm, which does change position with synthesis time, as shown in Figure 3A. To obtain a pure colloidal solution, dialysis is usually utilized. The dialysis process results in the loss of fluorescence signals in the performed experiment, as shown in Figure 3B, and high signals are found in the distilled water used for dialysis, indicating that there is a ratio of particles that are nonuniformly dispersed and are removed during the dialysis process.

**Figure 4:**
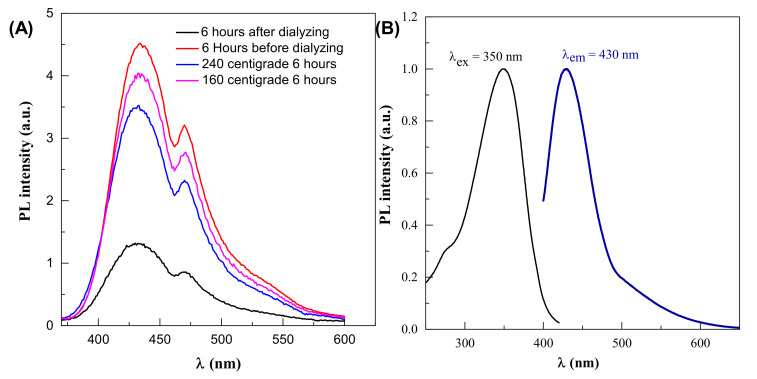
(**A**) Effect of dialysis on PL intensity (**B**) photoluminescence properties of CDs.

The optimal parameter for fluorescence signals in this reaction is 6 h synthesis time and 190 °C temperature, so changing these parameters in either way reduces the fluorescence intensity, as shown in Figure 4A. Dehydration of the precursor happens first, which causes decomposed products to aggregate and moderate condensation, resulting in large-sized nanoparticle polymerization. As the temperature rises, the polymer nanoparticles decrease due to continual intramolecular dehydration. Carbon bonds are created at this step, and aromatic clusters are formed inside the polymers at the same time. The nucleation of CDs occurs when the concentration of so-formed clusters approaches the crucial supersaturation point. Nuclei are formed during this step by the migration of aromatic clusters toward the particle surface, and different functional groups (carboxyl, hydroxyl, carbonyl, etc.) are simultaneously passivated. Polymer nanoparticles tend to go extinct (convert) over time, enabling CDs to take their place [37,38].

The quantum yield of the CDs was determined. The obtained results are shown in Figure 4B. The quantum yield (QY%) of the CDs is 6.3%, using 0.5 mg/mL Rhodamine B in ethylene glycol solution is used as reference.

### 3.2. DLS and Zeta Potential Analysis

**Figure 5:**
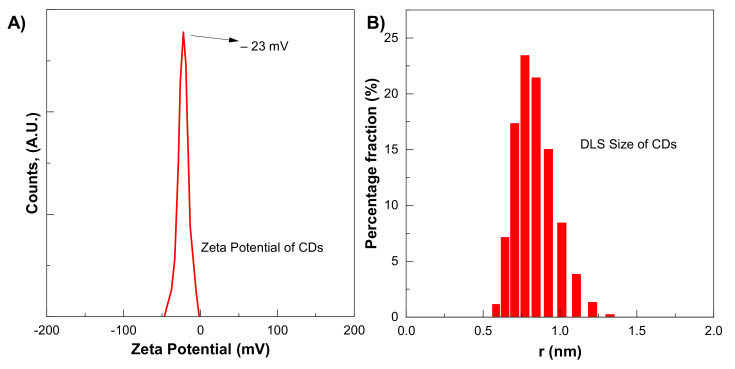
(**A**) Zeta potential (**B**) DLS Size of CDs for six-hour synthesis.

CDs size distribution and zeta potential were measured by zeta-seizer. The average CDs was 5.9 nm, which is to some extent larger than the TEM-measured size, as shown in Figure 5B. The DLS method measures the hydrodynamic diameter of the particle in the solvent, which is larger than the diameter in a vacuum. The presence of a large number of functional groups on CDs improves their dispersion in water-based solvents. The zeta potential of CDs shows a single peak centred at −23 mV, as shown in Figure 5A. The negative sign with zeta potential shows negatively charged residues that are essential for good dispersion stability of CDs.

### 3.3. TEM Analysis of CDs

**Figure 6:**
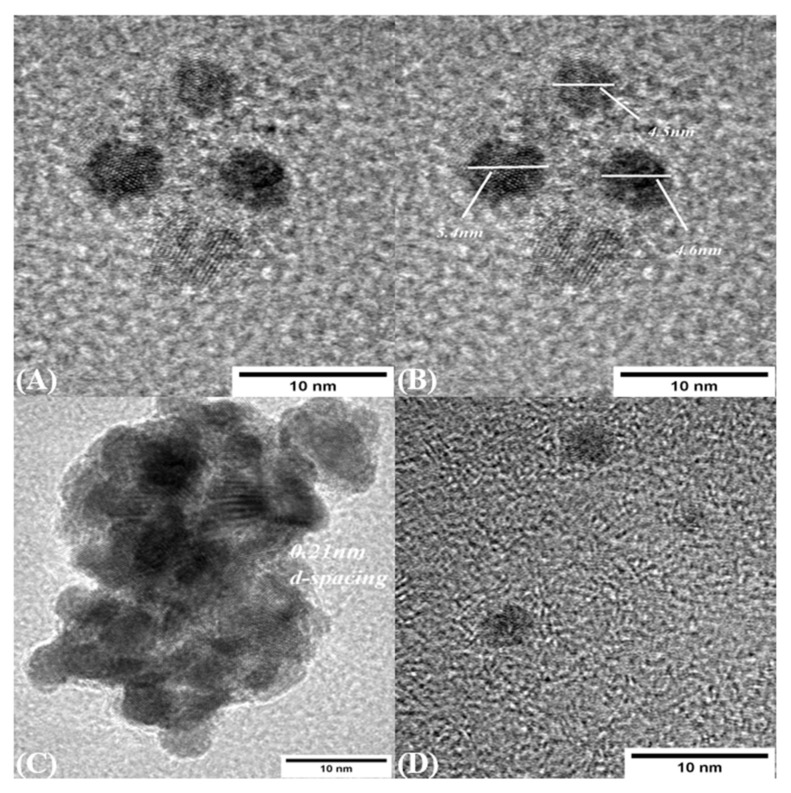
TEM images (**A**) Carbon dot presence (**B**) Size of Carbon dot in the range of 4–6 nm (**C**) Agglomerates of Carbon dot in the sample (**D**) Incompletes formation of carbon dot in the sample.

Size and morphology of CDs were measured using TEM and DLS. TEM images show that the CDs were synthesized in the range of 4–6 nm, which is consistent with the size measured from DLS, and were mostly dispersed nonuniformly. The interplanar spacing is measured as 0.21 nm, as shown in Figure 6A,C, which shows the agglomerate of the CDs which are formed during sample preparation and solvent preparation.

### 3.4. FT-IR Analysis of CDs and Ascorbic Acid

**Figure 7:**
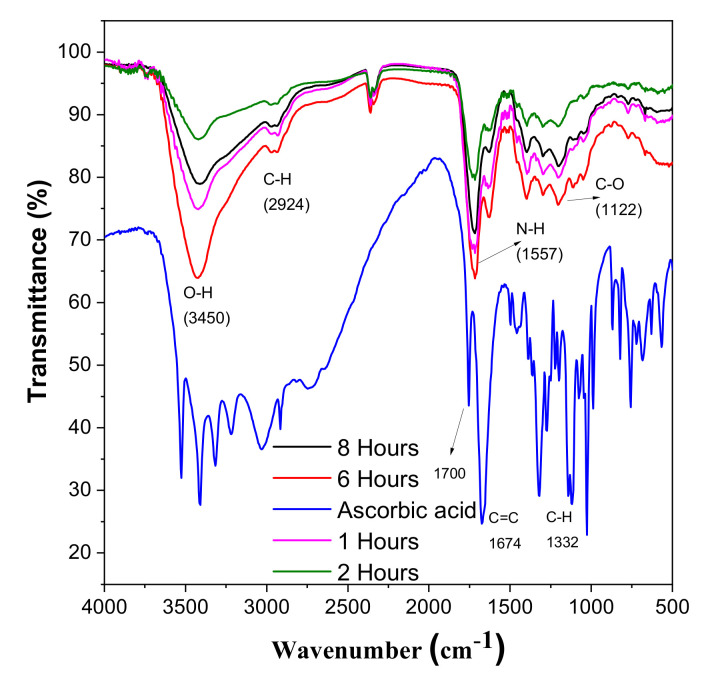
FTIR spectroscopy of CDs and ascorbic acid.

FT-IT spectroscopy was used to examine the changes in the chemical composition of L-ascorbic acid and CDs. FT-IR also helps to analyse the interaction between different species involved in the reaction. For the ascorbic acid, the small adsorption peaks near 3450 cm^−1^ represent different hydroxyl groups. The characteristic peak near 1750 cm^−1^ shows the stretching vibration of the C=O bond. After the hydrothermal reaction, these peaks vanished and a new peak which shows the formation of CDs appears in Figure 7 [39,40]. After the hydrothermal reaction, these peaks vanished and a new peak which shows the formation of CDs appears in Figure 7. The adsorption peak at 3450 cm^−1^ shows the presence of the carboxylic group [41]. One shared peak at 1557 cm^−1^ shows vibration and deformation bands of N-H, and a minor peak at 2250 cm^−1^ confirms this. There is one stretching vibration band at 1122 cm^−1^, which shows the presence of C-O [21,42].

### 3.5. XRD Analysys

**Figure 8:**
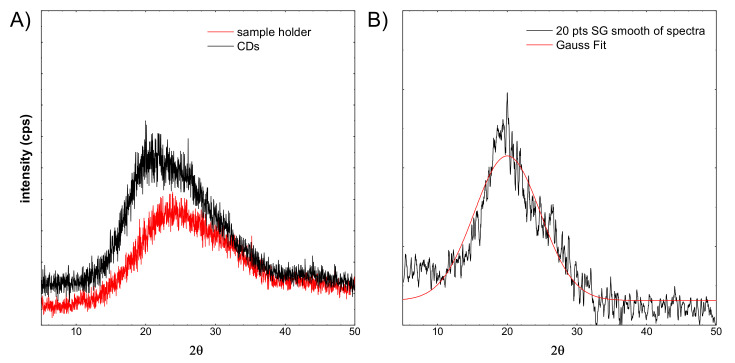
XRD spectra of: (**A**) sample holder and CDs, (**B**) the CDs spectrum after background subtraction and smoothing with a filter.

XRD measurements were performed. The obtained results are shown in the figure below (see Figure 8).

Figure 8A shows a red line registered for the sample holder. Typically, the glass exhibits a very wide peak around 25°. The sample CDs were imposed on a glass sample holder. The spectrum shift is visible. It is related to the overlapping of two components. The first comes from glass, the second probably from the CDs structure. From the recorded CDs spectrum, the background spectrum was subtracted (see Figure 8B), then smoothed out. Subsequently, the Gaussian curve was fitted to the spectrum. This fitted peak is apparently very broad. Taking into account the Scherrer equation, the crystallite size was calculated. In this case, it is equal to 0.89 nm. This is in line with the HR-TEM observations, and the observed particles are not single crystals.

### 3.6. Metal Ions’ Detection and Quantification

**Figure 9:**
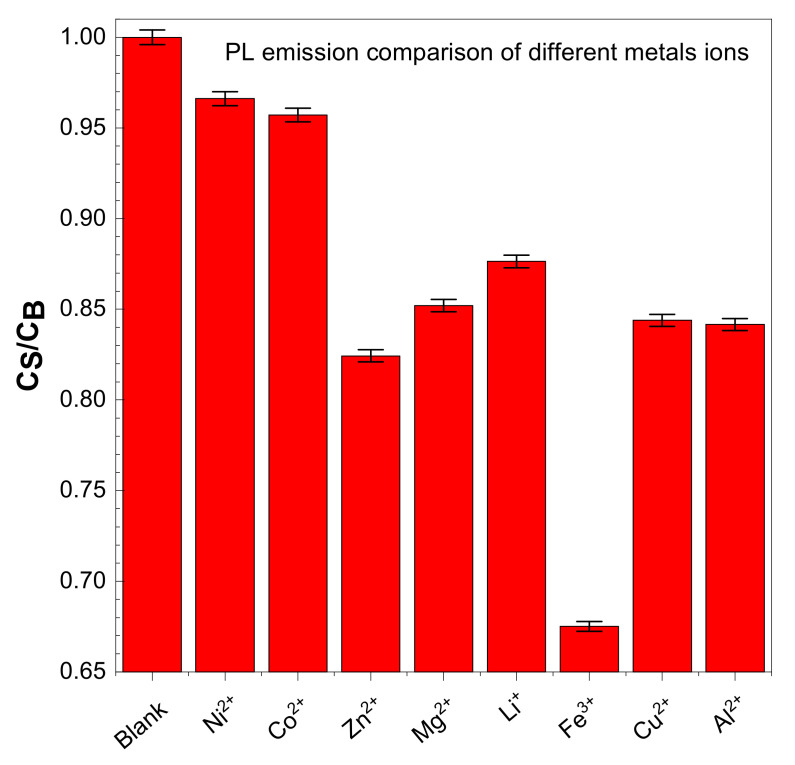
Effect of PL spectra ratio of carbon dots during sensing with different salts.

CDs have good water solubility, and the metal ion detection ability of CDs is measured by adding different metals salts in a water solution of CDs. Typically, 100 µL of salt/2.9 mL of CDs solution was used to obtain the PL spectra. The following metal ions were used: ZnCl_2_, CuCl_2_, Mg(ClO_4_)_2_, NiSO_4_·7H_2_O, CoSO_4_·7H_2_O, Al(ClO_4_)_3_·9H_2_O, LiClO_4_, and Iron(III) nitrate nonahydrate Fe(NO_3_)_3_·9H_2_O. The solution was excited at 350 nm to obtain PL spectra. All the salts that we tested do not show variation in their PL spectra, but for the Fe^3+^ there are significant decreases in PL spectra—up to a 40% reduction, as shown in Figure 9.

The presence of hydroxyl and carboxyl groups was confirmed from the FTIR in the CDs. Fe^+3^ can form a stable complex with CDs through carboxyl functional groups, resulting in the quenching of fluorescence. This is described as high selectivity of CDs to Fe^3+^ and electron transfer based on the photoinduced method [2,43].

Various types of metal salts are used in the existing research, including nitrates, sulphates, perchlorates, and chlorides. Therefore we examined the effectiveness of CDs on metal ion detection.

The smallest quantity or concentration of the analyte in the test sample that can be reliably differentiated from zero is known as the limit of detection (LoD), and the lowest concentration of analyte that can be measured with satisfactory reproducibility and trueness is known as the limit of quantitation (LoQ) [44,45]. We took seven measurements to determine the concentration. Table 1 shows the values of normalized intensity values of emission spectra with a corresponding concentration level of salt. WeIt is not possible to extrapolate LoQ; it must be calculated using the appropriate standard measurement or reference sample. We used the standard deviation method to find these quantities [31,45]. The formulas for LoD and LoQ are the following:

(1) $$LoD=3.3\times \frac{\sigma }{S}$$

(2) $$LoQ=10\times \frac{\sigma }{S}$$

where:

*σ* = Standard deviation of the results;

*S* = Slope of the curve.

**Table 1:** Values of concentration of salt and corresponding intensity values of emission spectra.

The Concentration of Fe^3+^ ions, (ppm)	(Fo − F)/F, (a.u.)
0	0
1	0.047297
5	0.091478
10	0.173918
20	0.224868
30	0.294106
60	0.39436

To find the *LoD* and *LoQ*, we used 1000-times diluted CDs with different concentrations of Fe^+3^ salt. The pH of the quantum dots, as well as salt, was set to 1 using HClO_4_. Thanks to that, the effect of pH on the photoluminescence of QDs was eliminated. We first found the calibration curve in the form of y = mx + c, which is y = 0.0062x + 0.0632, and R^2^ = 0.8992. The parameters were obtained from the data given in Table 1. The standard deviation of the slope was calculated as 0.000931. Furthermore, x is the concentration of Fe^3+^ and c is the intercept value, and these values are used in Equations (1) and (2) to find *LoD* and *LoQ*, respectively.

**Figure 10:**
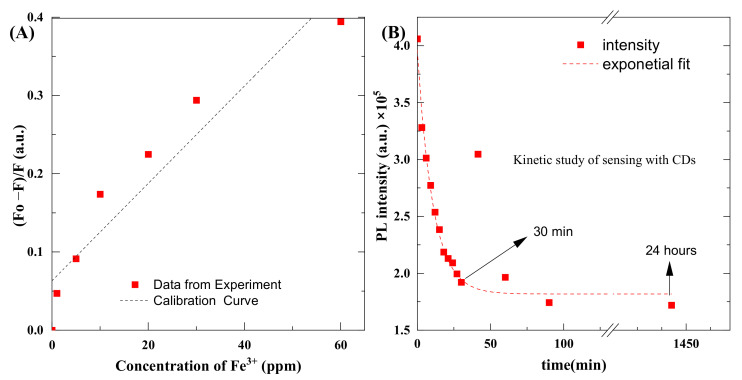
(**A**) Concentration vs. the intensity of emission spectra, (**B**) kinetic plot of the reaction between CDs and Fe^3+^.

Typically, 2.9 ml of CDs solution was mixed with 0.1 mL of salts, and we measured the effect of different concentrations of salts on emission spectra of the CDs. Measurements were recorded 1 min after mixing the reagents. Figure 10 shows the plot of concentration and intensity of emission spectra. The value of LoD was found to be 0.49 ppm and the value of LoQ was 1.48 ppm.

Some deviation of points is observed. Originally, we assumed this might be related to pipetting accuracy. However, once the expected dilution error was calculated, the observed differences could not be explained. That is why we conducted kinetic studies. Figure 10B shows kinetic plots of photoluminescence that decrease as a result of a chemical reaction between CDs and Fe^3+^ ions. It should be noted that a further decrease in photoluminescence, from a practical point of view, means an increase in the sensitivity of the proposed method. Detection efficiency enhances with time, and it increases rapidly up to 30 min and then remains consistent, even after 24 h. From the measurements, it can be observed that detection efficiency of CDs was increased up to 200% after 30 min, and this kinetic study will be part of further studies.

This kinetic curve explains the uncertainty observed during the preparation of the calibration curve. A slight delay in the measurement or calibration point can cause a significant change in the readings. The fitted kinetic curve corresponds to the first-order reaction. Further kinetic studies will be the subject of further studies. This confirms that there is a relationship between Fe^3+^ ions and CQDs. So far, there are no reports in the literature about the mechanism of this reaction.

## 4. Conclusions

The paper presents a simple, eco-friendly method for the synthesis of CDs. Vitamin C was used as a precursor. It has been shown that by changing the synthesis time of nanoparticles, it is possible to obtain different emission spectra. The longer the synthesis time, the longer the wavelength the particles emit. The obtained particles are characterized by high quantum efficiency. Thanks to the possibility of synthesizing very high-grade particles (100 g/L), PL is visible even in daylight. Spectacularly, intense blue colour can be observed in the dark after excitation with light of 350 nm.

Further study has been carried out on the application of the obtained quantum dots to determine the content of Fe^3+^ in an aqueous system. Research confirms that among the selected metal cations (and anions, since different types of metal salts were tested, nitrates, sulphates, perchlorates, and chlorides), only the presence of Fe^3+^ reduces the PL of the solution. Therefore, as-synthesized CDs are a good contender for the detection of Fe^3+^ in an aqueous system. Research was also carried out to find the LoD and LoQ of the detection system. It was observed that after 30 min, detection ability of CDs increased 200%, so it is recommended to use this length of time to optimize detection. The efficiency of detection will increase with time, and this will be investigated in the continuous research carried out on the topic. Further research will also be carried out on the use of these particles to detect traces of blood at crime scenes, instead of the toxic and dangerous luminol.

## Data Availability

Not applicable.
